# Attention-Based Convolutional Neural Network for Ingredients Identification

**DOI:** 10.3390/e25020388

**Published:** 2023-02-20

**Authors:** Shi Chen, Ruixue Li, Chao Wang, Jiakai Liang, Keqiang Yue, Wenjun Li, Yilin Li

**Affiliations:** School of Electronic Information, Hangzhou Dianzi University, Hangzhou 310005, China

**Keywords:** artificial intelligence, ingredients identification, convolutional neural network, multi-attention module, open set recognition

## Abstract

In recent years, with the development of artificial intelligence, smart catering has become one of the most popular research fields, where ingredients identification is a necessary and significant link. The automatic identification of ingredients can effectively reduce labor costs in the acceptance stage of the catering process. Although there have been a few methods for ingredients classification, most of them are of low recognition accuracy and poor flexibility. In order to solve these problems, in this paper, we construct a large-scale fresh ingredients database and design an end-to-end multi-attention-based convolutional neural network model for ingredients identification. Our method achieves an accuracy of 95.90% in the classification task, which contains 170 kinds of ingredients. The experiment results indicate that it is the state-of-the-art method for the automatic identification of ingredients. In addition, considering the sudden addition of some new categories beyond our training list in actual applications, we introduce an open-set recognition module to predict the samples outside the training set as the unknown ones. The accuracy of open-set recognition reaches 74.6%. Our algorithm has been deployed successfully in smart catering systems. It achieves an average accuracy of 92% in actual use and saves 60% of the time compared to manual operation, according to the statistics of actual application scenarios.

## 1. Introduction

The catering industry is one of the country’s leading industries [1,2]. Government agencies, private restaurants, and school units are all inseparable from catering. In recent years, with the development of technologies, artificial intelligence has been being used almost everywhere and is considered a core skill for the future. The AI market is projected to grow to $190 billion by 2025 [3]. Under such a trend, smart catering [4] combining artificial intelligence with traditional catering is becoming increasingly popular. Although the descriptions of artificial intelligence are various, the core of it is widely believed to be the research theories, methods, technologies, and applications for simulating, extending, and expanding human intelligence [5]. Integrating these advanced technologies with the catering industry has become a research hot spot [6]. Its main advantages are as follows: (1) Using digital technology, digital thinking, and digital cognition to promote the digital transformation and intelligent upgrade of the industry; (2) Using artificial intelligence [7] to replace traditional manual labor and significantly reduce labor costs; (3) Forcing deep-level systemic system remodeling [8], promoting fundamental changes in management processes and rules; (4) Relying on the “Ingredient Standard Acceptance Pictorial Database”, the data-based and standardized management of the whole process of food bidding procurement, acceptance inspection, supplier and personnel assessment, to achieve the purpose of system optimization and management innovation. The acceptance test of ingredients is the first and crucial step in the entire catering workflow. At present, almost all catering companies need staff to distribute and inspect the ingredients provided by the suppliers, which is time-consuming and lacks uniform standards. Therefore, many researchers are committed to realizing intelligent catering with the help of automated identification technology, to improve distribution efficiency and reduce the use of manpower. As a result, quickly and accurately identifying the ingredients becomes a critical part of smart catering.

Currently, the ingredients identification approaches can be roughly divided into two categories: traditional manual feature extraction methods and deep learning methods. For example, He et al. [9] extracted the global and local features of the food image using methods such as K-nearest neighbors and vocabulary tree to achieve 64.5% accuracy in 42 types of cooked food classification; Nguyen et al. [10] proposed a classification method using local appearance information and global structure information, achieving 69% accuracy in the classification of six kinds of foods; Farinella et al. [11] achieved the accuracy of 67.9% in the 61 classifications of the Pittsburgh fast food image dataset based on the Bag of Textons image representation method combined with the support vector machine (SVM); Joutou et al. [12] introduced multi-core learning to extract various image features such as color, texture, and scale-invariant feature transform (SIFT) to achieve the accuracy of 61.34% in 50 kinds of food. Liao et al. [13] used deep learning methods and the maximum class spacing loss function to recognize food images with an accuracy of 69.2%; Wu et al. [14] used a deep learning algorithm based on domain confusion and prior trees combined with order information to achieve an accuracy of 72.45% in the classification of 60 kinds of fresh food ingredients.

From the above research status, it can be seen that most studies focus on cooked food rather than fresh ingredients, and even the identification research involving fresh ingredients only involves vegetables and fruits. These studies still have some shortcomings: (1) The lack of an applicable large-scale ingredients database. (2) The accuracy of ingredients recognition is low. (3) They cannot deal with new categories outside the database. To overcome the problems above, in this paper we build a large-scale database and design an end-to-end attention-based DenseNet [15] model with an open-set module. The attention module can help improve accuracy by reducing the background interference in the pictures. The open-set module can deal with new categories outside the database.

The main workflow of our algorithm is as follows. First, we obtain images from different application scenarios and name the ingredients according to a unified naming standard. After data cleaning, we establish a large-scale database that contains more than 65,000 pictures of ingredients and conduct the data augmentation. Second, we design a DenseNet model with a multi-attention module to help extract features more effectively. The attention module includes a channel module and a spatial module and helps the model focus on crucial information. Then, we input the images into the network for training and obtain a classification model. To deal with new categories outside the database, we add an open-set module after the classification network to get the prediction result in the open-set condition. The open-set recognition [16] module is relatively independent so it does not affect the primary network model. 

The main contributions of this paper are:

(1) Constructing a large-scale fresh food image database for common ingredients in China for the first time.
(2) Building an end-to-end multi-attention based DenseNet model for ingredients identification and achieving high accuracy.
(3) Applying open-set recognition for the first time in the field of ingredients recognition to help solve the problem of new categories in practical applications.

The contents of each part of this paper are arranged as follows: Section 1 is an overview of this paper, explaining the research background; Section 2 is experimental data and methods, introducing the construction process of our ingredients database and the whole network model; in Section 3, experimental results show the performance of our model in the ingredients recognition task; and the discussion and conclusion are drawn in Section 4 and Section 5, respectively.

## 2. Materials and Methods

### 2.1. Database Building 

#### 2.1.1. Images Collection

**Figure 1:**
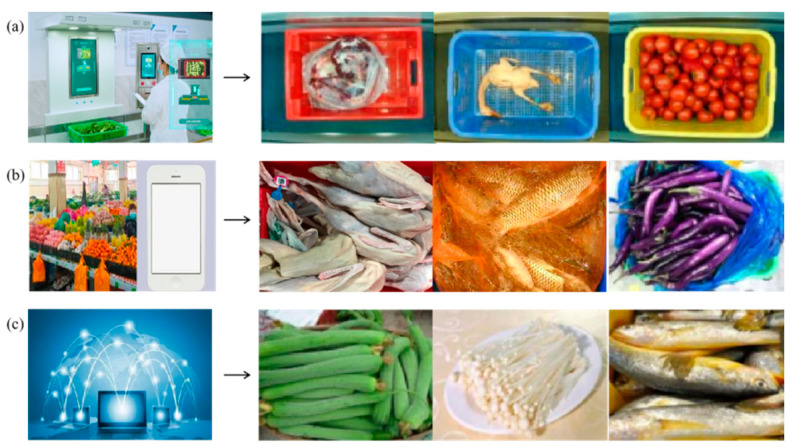
Examples of images from different sources. (**a**) Images from enterprises and schools taken by our device. (**b**) Images from market taken by mobile phones. (**c**) Images from the Internet.

In order to better ensure the generalization of the algorithm, it is necessary to ensure the diversity of data as much as possible. In this paper, we consider the ingredients’ source, image taking equipment, shooting season, and ingredients state during the image acquisition. To ensure the samples’ diversity, we choose four scenarios for data collection: catering companies, school canteens, farmer’s markets, and the Internet. Examples of pictures collected in different ways are shown in Figure 1. In catering companies and school canteens, we install a smart device with cameras to take pictures of the ingredients, as shown in Figure 1a. The shooting resolution is uniformly set to 800 × 600. The ingredients are placed in random-colored baskets. During the shooting process, proper manual operations are adopted to increase diversity such as placing the ingredients in different positions of the frame, changing the relative position between the ingredients, and changing the quantity of the ingredients. For some ingredients that may have packaging bags, both pictures with and without the original packaging are collected. In the market, as shown in Figure 1b, we use mobile phones to get more pictures with different lighting, angles, and degrees of freshness. This work lasts for one year to ensure that pictures of those seasonal solid ingredients can be collected. Additionally, we collect copyright-free ingredients pictures from the Internet, as shown in Figure 1c. These pictures usually have more complicated backgrounds, which can further increase the diversity of samples. Finally, a large-scale food data set with more than 400 types of ingredients is obtained, containing 68,637 images. The statistics of the food data set are as Table 1.

#### 2.1.2. Ingredients Naming

**Figure 2:**
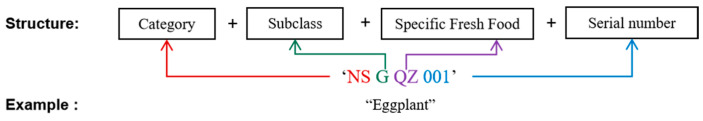
An example of naming an image using our method.

One of the purposes of this paper is to build a large-scale fresh food database. We first formulate a set of unified naming standards referring to national standards. We use the English letter plus number naming method to classify the foods in multiple levels. The naming consists of four parts. The first part is category, including non-staple food (NF), meat and poultry (NR), fruits and vegetables (NS), and aquatic products (NX). The second part is the subclass under the category. For example, in Figure 2, ‘G’ represents the rhizomes class under ‘NS’. The third part is the specific class represented by Chinese abbreviations. The last part is the serial number of the specific image. For example, the first picture of the class eggplant is named NSGQZ001, as shown in Figure 2. All the fresh food ingredients in the library are named in this way. This naming method can reflect the degree of differentiation between different ingredient categories and are conducive to statistical results.

### 2.2. Images Augmentation

Since the resolution and size of pictures from different sources are not the same, we apply a unified re-sampling operation to process the pictures to the same resolution. We tried various resolutions during the model training process and found that the accuracy was the highest when the resolution was set to 600 × 600, indicating that the network can extract more useful information at this resolution. The accuracy at different resolutions is shown in Table 2.

**Figure 3:**
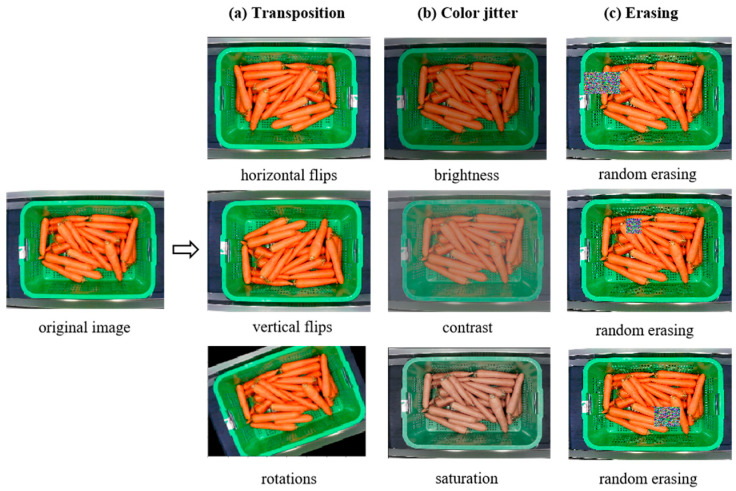
Examples of data augmentation.

On the other hand, we apply a variety of methods to realize data augmentation, including transposition, color jitter and random erasing, as shown in Figure 3. 

As shown in Figure 3a, the transposition method includes random flips and rotations. The translation range is from 100 pixels to −100 pixels in the horizontal and vertical directions. The rotation angle was between 90 degrees and −90 degrees. The number of images increases three times after transposition methods. As shown in Figure 3b, the color jitter method includes random changes in brightness, contrast and saturation. During training, the brightness and contrast enhancement factors are randomly between 0.5 and 1.5, and the saturation enhancement factor is randomly between 0 and 2. After color jitter methods, the number of images increased three times. As shown in Figure 3c, the random erasing scale is randomly between 0.02 to 0.05. After erasing methods, the number of images increased two times. Finally, after data augmentation, we obtain a data set which is 18 times the original one. 

### 2.3. Attention-Based Convolution Neural Network

**Figure 4:**
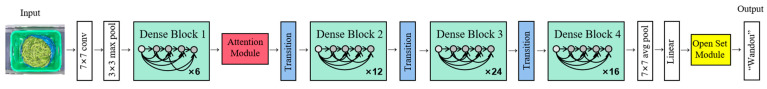
The overall structure of our network. The green frame represents dense blocks, the red frame represents attention module, the blue frame represents transition layer and the yellow frame represents open set module.

The classification algorithm network designed in this paper is inspired by the structure of the DenseNet model, and we add a multi-attention module. The overall structure is shown in the Figure 4.

#### 2.3.1. Densely Connected Convolution Network

DenseNet fully applies the idea of cross-layer connection to every single layer in the module, which means the input of any convolution layer contains the output of all convolution layers before. This structure can integrate high and low-level features so that the features can be fully reused, which effectively suppresses over-fitting and reduces the number of parameters. The core of the DenseNet structure is the dense block and the transition layer. 

The dense block is a densely connected module and the transition layer is the connection area between two adjacent dense blocks. The connections inside each dense block are shown in Figure 4. The processing of input features can be expressed as:

(1) $$X_{l}=H_{l}\left(\left[X_{0},X_{1},\ldots ,X_{l-1}\right]\right)$$

where $X_{0}$ is the input, H is the nonlinear conversion function including three operations of batch normalization (*BN*) [17], linear rectification function (*ReLU*) [18] and two convolution layers with the kernel size of 1 × 1 and 3 × 3, respectively. $X_{l}$ represents the output of the lth layer. 

The BN normalizes the data to meet the standard normal distribution, so it reduces the calculation time of the entire training set gradient. The calculation method is as:

(2) $$y_{i}^{\left(b\right)}=BN{\left(x_{i}\right)}^{\left(b\right)}=\gamma \cdot \left(\frac{x_{i}^{\left(b\right)}-\mu \left(x_{i}\right)}{\sqrt{\sigma {\left(x_{i}\right)}^{2}+\varepsilon }}\right)+\beta$$

where $x_{i}^{\left(b\right)}$ represents the value of the ith input node of the layer when the input is the bth sample of the current batch, $x_{i}$ is a row vector composed of $\left[x_{i}^{\left(1\right)},x_{i}^{\left(2\right)},x_{i}^{\left(3\right)},\ldots ,x_{i}^{\left(m\right)}\right]$ and the length is the batch size value m. μ and σ represent the mean and standard deviation, respectively. ε is the minimal amount introduced. γ and β are the scale and shift parameters of the row. $y_{i}^{\left(b\right)}$ is the normalized result. We choose to use *ReLU* as activation function. Comparing to other activation functions such as Sigmoid function and Tanh function, *ReLU* can make the process of gradient descent and error back propagation more efficient, and at the same time avoid the problem of gradient disappearance. The *ReLU* function is a piece-wise linear function, which turns all negative values into 0, while the positive values remain unchanged, which is a one-sided suppression. When the input is a negative value, the neuron will not be activated, that is, only some neurons will be activated at the same time, which makes the neurons in the neural network have sparse activation, thereby speeding up the calculation efficiency.

**Figure 5:**
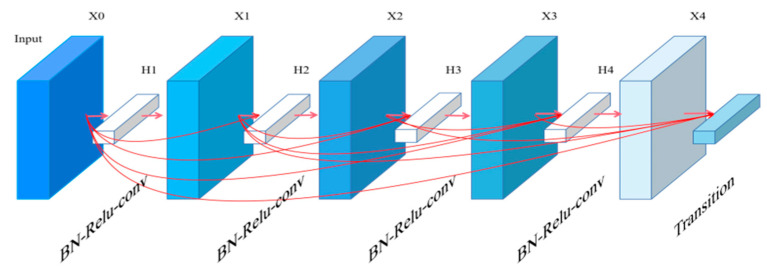
The inside connection structure of dense block.

The transition layer (as shown in Figure 5, white box) between every two dense blocks contains three parts: BN, convolution layer and average pooling layer. Its application is mainly to reduce the output feature dimension of dense blocks and improve the calculation efficiency. At the same time, the transition layer can achieve feature down-sampling. Among the transition layers, the kernel size of convolution is 1 × 1, and it can realize feature channel dimension reduction. The 2 × 2 average pooling layer can realize feature down-sampling. The relevant parameter of channel dimension reduction is the compression rate θ that represents the dimension reduction proportion, the value of which is from 0 to 1. 

We use DenseNet121 as our backbone model, which contains four dense blocks, as shown in Figure 5. The number of dense layers contained in each dense block is 6, 12, 24, and 16, respectively. The channel parameters of the input feature are set to 64. Through the operation of the convolution layer, the images input are down-sampled and dimensionality reduced. 

#### 2.3.2. Multi-Attention Module

Since the background of different images is different, it is crucial to make the network pay more attention to the main parts of the picture and extract more valuable characteristics.

**Figure 6:**
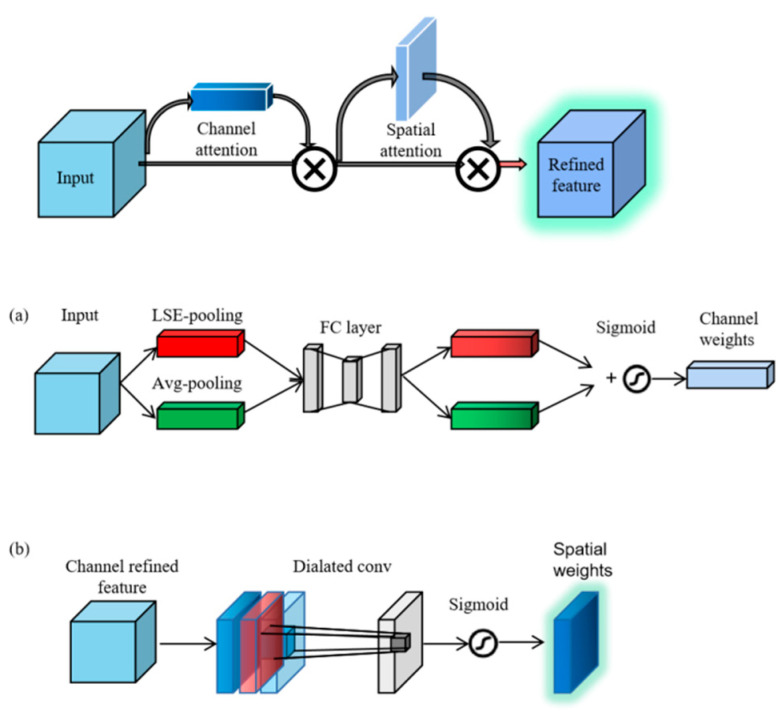
The structure of the multi-attention module. (**a**) The channel attention module. (**b**) The spatial attention module.

We design an improved multi-attention mechanism based on CBAM [19], including two relatively independent sub-modules: channel attention [20] and spatial attention modules. The structures of these two attention modules are shown in the top picture in Figure 6. After operations such as convolution, the amount of information contained in different channels of feature maps is different. The channel attention mechanism can assign more weight to the channels that contain more helpful information. The spatial attention mechanism focuses on learning the position information of the picture and can assign different weights to different areas so that the network pays more attention to the functional area. In this paper, we add the multi-attention module after the first dense block. Furthermore, the composition order of the entire attention module is that the channel attention module is in the front, and the spatial attention mechanism is in the back. The module’s input is the feature map extracted by the first dense block. The weight matrix obtained by the channel attention module is multiplied by the original input to obtain the optimized output feature map and then input to the spatial attention module. After another weight optimization, the improved feature map is input to the transition module to continue the follow-up process.

In the channel attention module, as is shown in Figure 6a, we use LSE pooling layer [21] instead of Max pooling layer, which is calculated as:

(3) $$x_{p}=\frac{1}{r}\cdot \log\left[\frac{1}{S}\cdot \sum \left(i,j\right)\in S^{\exp\left(r\cdot x_{ij}\right)}\right]$$

where $x_{ij}$ represents the activation value at the pixel. S=s×s is the total number of points in the pooling area S, and r is the hyper-parameter. We set the value of r to 2.

In the spatial attention module, as is shown in Figure 6b, we replace the original 7 × 7 convolution kernel with a dilated convolution [22] kernel of 5 × 5 and the interval of the convolution kernel is set to 1, which increases the receptive field and reduces the amount of computation.

### 2.4. Open-Set Recognition Module

The above algorithm might encounter two main problems after being deployed in actual application scenarios: (1) Some types of existing ingredients appear infrequently, and it is hard for users to collect enough pictures in a specific period time. If these kinds of food are added to the training set, the difference in the number of samples will be too large, which might cause model bias. (2) With the change of seasons, demand, and other factors, the types of food supplied every day are not static, and new categories of ingredients outside the data set will appear. 

**Figure 7:**
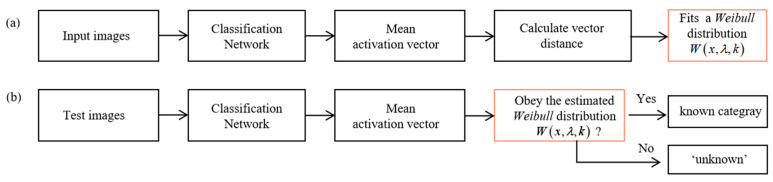
The workflow of the open set module. (**a**) The training phase. (**b**) The prediction phase.

To solve the above problems in practical applications, we introduce an improved open-set recognition module [23] based on OpenMax [24] into our model. The main advantages of this module are as follows: (1) The feature extraction capability of the previous network is still valid, and we only need to add a small amount of computation. (2) It is relatively independent and we can flexibly select whether to use it or not, without affecting the original identification of categories. The main workflow is shown in Figure 7. It consists of two phases: the training phase and the prediction phase. During the training phase, we fit a *Weibull* distribution $W\left(x,\lambda ,k\right)$ [25] using the activation vectors extracted by the model for each category. In the prediction phase, it calculates the distance between feature vectors of the input image and existing categories, and judges if it obeys the known distribution $W\left(x,\lambda ,k\right)$. If it is, the input image will be predicted to be a known category; otherwise, it is predicted to be ‘unknown’.

We have conducted many open-set recognition tests for the specific needs of multiple different application scenarios. The data differences in different scenarios are mainly the total number of categories and openness. Openness is used to measure the degree of an open set and its calculation equation is as:

(4) $$Openness=\frac{k_{unknown}}{k_{all}}$$

where $k_{unknown}$ represents the number of new categories outside the data set and $k_{all}$ represents the number of categories in the existing data set. According to actual investigation, the openness in application scenarios is about 0.2–0.4.

## 3. Experiments and Results

### 3.1. Experiment Setup

Our experiment was carried out on a workstation with four NVIDIA GEFORCE RTX-2080Ti GPUs and four Intel Xeon Silver 4110 CPUs. The memory of each GPU is 11 GB. The operating system for training models is Ubuntu 16.4. The deep learning framework used in training models is PyTorch GPU.

After investigating the occurrence of daily ingredients in different scenarios, we select 170 categories with the highest frequency of occurrence for training to ensure that the number of pictures of each category is sufficient, containing 31,200 pictures. The average number of pictures in each category is 184. After data cleaning, we remove invalid images that are too similar and obtain a database containing 25,000 pictures. We randomly divide the images into the training set, the validation set, and the test set with ratios of 56.25%, 18.75%, and 25%. The number of pictures in the training set is 14,062, the number of pictures in the validation set is 4688 and the number of pictures in the test set is 6250. The remaining unused pictures can also be used for testing.

The optimizer we apply is stochastic gradient descent (*SGD*) [26] and the loss function is cross-entropy loss. The initial learning rate is set to 0.001, and it can be automatically adjusted during the training process according to the learning rate decaying method. The batch size is set to 8.

### 3.2. Performance Evaluation 

The evaluation indicators applied in this paper are accuracy, recall, precision and *F*1 value. The calculation method is as:

(5) $$accuracy=\frac{T}{R}\;recall=\frac{1}{n}\sum_{i=1}^{n}\frac{TP_{i}}{R_{i}}\;precision=\frac{1}{n}\sum_{i=1}^{n}\frac{TP_{i}}{TP_{i}+FP_{i}}\;F1=\frac{2\times recall\times precision}{recall+precision}$$

where T represents the number of correctly identified samples in the sample to be tested, and *R* represents the total number of samples to be tested. $TP_{i}$ represents the number of correctly identified samples of the ith class in the sample to be tested, $R_{i}$ represents the total number of samples of the ith class in the sample to be tested, and *n* is the total number of categories. $FP_{i}$ represents the number of non−i−type samples identified as the ith type in the sample to be tested.

### 3.3. Classification Results

#### 3.3.1. Non-Open Set Recognition

**Figure 8:**
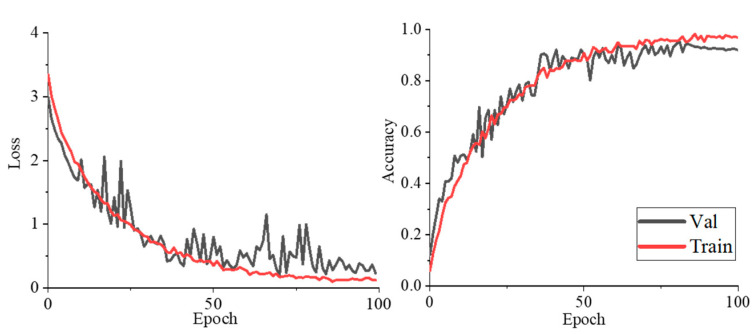
The loss and accuracy changing conditions during the training process.

We first perform a classification test in non-open data set cases. The change curve of the loss and accuracy values of the training set and validation set with the epoch during the training process is shown in Figure 8. It shows that the loss gradually decreases with the increase of training times, and the accuracy gradually increases. After 70 epochs, the corresponding curves of each network tend to be flat. The accuracy of the training set is slightly higher than that of the validation set, indicating that there is no over-fitting during the training process. The accuracy of our algorithm is 97.72% for the training set, 96.29% for the validation set and, 95.90% for the testing set.

**Figure 9:**
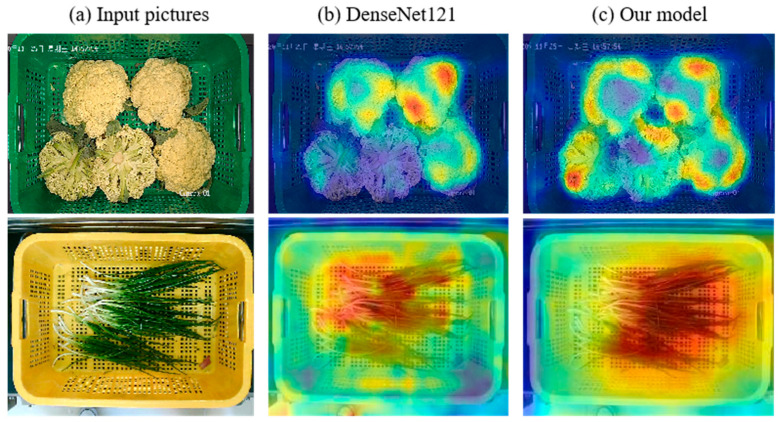
Visualization results of different network. (**a**) The input image of the model. (**b**) The visualizing feature map of DenseNet121 model. (**c**) The visualizing feature map of our model.

To further illustrate the effect of the attention module in our model, we use Grad-CAM [27] method to visualize the output of the last layer of our model after inputting a single image, as Figure 9 shows. This method can intuitively reflect the network’s attention to different parts of the input image. Figure 9a is the original input pictures. Figure 9b,c are the visualizing results of a DenseNet121 model and our approach, respectively. The comparison shows that the attention module can help our model focus on the major parts of the whole image, which means that our network can better avoid the influence of interference factors such as the background.

#### 3.3.2. Open-Set Recognition

We count the order information of ingredients in several application scenarios and find that the openness is around 0.2–0.4. In this paper, we use several open-set conditions to test the effectiveness of our model. For example, in one of the conditions, there are 60 common categories. Among them, 6 categories are outside the data set and 9 categories are in the data set but with a low frequency of occurrence. We combine these 15 categories and consider them unknown categories. Another 45 categories of ingredients are used for training, including 2600 images. The openness of this condition is 0.25. We improve the open set module for our data by using a combination of cosine distance and standardized Euclidean distance to represent the category difference, and the weights of the two are 0.8 and 0.2, respectively. During the training process, the first round of distribution fitting is carried out after 30 epochs to ensure enough effective activation vectors. After that, we update the distribution model every 30 epochs. The test set includes all 60 kinds of food. When inputting existing food images, the output should be the correct category. Otherwise, the result is incorrect; the output should be “unknown” when inputting unknown food images. In Table 3, we summarize the performance of our model in different open set conditions and compare it with the threshold method. The threshold method only sets a likelihood threshold to determine whether the input image is an unknown category. Table 3 shows that among all the trials, the recognition accuracy of our model in open set conditions is 6.1% higher than the threshold method on average.

## 4. Discussion

To select a suitable model as the backbone network, we use a variety of models for experiments. The results are shown in Table 4. It can be seen that DenseNet121 achieved the best accuracy among all the models and with the smallest parameter amount. So we choose DenseNet121 as our backbone network. 

Since DenseNet121 has four densely connected blocks, in order to get the best structure, we added attention modules in different positions and conducted multiple sets of experiments. The model situation and accuracy performance are shown in Table 5. The number after DenseNet121 represents the place where we added the attention module. For example, DenseNet121-1 means that the attention module is after the first dense block. ‘d’ represents our use of dilated convolution in the attention module and ‘lse’ represents our use of the LSE pooling layer. It can be seen from Table 5 that the attention module can improve the accuracy in most of the conditions and DenseNet121-1 has better performance, while the accuracy decreases after the attention module is added to all four blocks, even lower than the ordinary DenseNet121. A possible reason is that the excessive weight distribution mechanism reduces the effectiveness of the features extracted by the network. Hence, we continued to improve the network based on DenseNet121-1. Our model achieves the highest accuracy, 95.90%, among all the experiments. It can be seen from the number of parameters that the CBAM module is a lightweight module, and will not add too much complexity while improving the accuracy.

It is noted that under the same structure, the algorithm based on the shallower network model and the algorithm based on the deeper network model have little difference in accuracy performance, indicating that the model has the possibility of further compression and simplification. Since our algorithm is ultimately deployed on application devices, simplifying the model is of great significance for improving computing speed and saving costs. We will continue our work on model compression in the next stage.

We summarize the performance of our model and other related studies in Table 6. It shows that the scale of our database is very large. Although the data set of Hou et al. [30] contains more images, it only contains vegetables and fruits and a large part of the images come from the corresponding categories in Imagenet [31] data set. These images are not suitable for application scenarios. Our data set includes meat, seafood and other categories so the richness of data is much better. Our model’s recognition accuracy rate reaches 95.90%, which is much higher than other algorithms under the same order of magnitude of data. At the same time, the recognition speed of a single image is less than 0.2 s, which is suitable for deployment in application scenarios. 

Our algorithm has been used in 4 application scenarios and the average accuracy reaches 92% according to statistics, which illustrates the generalization of our algorithm. However, the accuracy is 3.9% lower than the performance under the test set. We analyze that the reason for this result is that there are differences in the distribution of ingredients and pictures in different application scenarios. In the future study, we will add new images taken during the use of the algorithm to the data set and update the recognition model to further improve the accuracy and generalization performance of the algorithm.

In this paper, we apply open set recognition to the field of fresh ingredients recognition for the first time to solve the recognition problem of new categories in actual scenarios. The setting of openness degree and the total number of categories in the experiment is based on the actual situation of the scene. The recognition results in Table 3 show that the recognition accuracy is relatively high when the openness degree is small. And the accuracy is related to the total number of categories participating in the training and the specific category when the open set degree is the same. At present, there is still room for improvement in the accuracy of open set recognition in practical applications. We speculate that open set recognition may require a larger data set. In future work, we will also try other open-set recognition methods such as G-OpenMax [34] and CROSR [35].

## 5. Conclusions

Quick and efficient identification of ingredients is the first and crucial step in the smart catering workflow. This paper mainly solves the problem of high-accuracy automatic recognition of common fresh ingredients in China, which effectively improves efficiency and accelerates the promotion and popularization of smart catering.

In this paper, a large-scale fresh ingredients data set was constructed. Our data set includes most of the common ingredients in China, and has strong universality and value for research in this field. An end-to-end multi-attention-based convolutional neural network model for ingredients identification was proposed and achieved an accuracy of 95.90% in 170 kinds of classification. The result is better than other related research studies. The multi-attention mechanism and the adjustments we made according to the specific conditions of our data set enable the network to extract more valuable features and effectively improve the recognition accuracy. At the same time, our algorithm has been deployed in many catering-related enterprises. The average accuracy in different practical application scenarios is 92%, which illustrates that our algorithm has good generalization capability. To solve the problem of new categories in practical application scenarios, we apply open set recognition in ingredients recognition for the first time. An improved OpenMax module is added to the network and achieves an accuracy of 74.7% in the open set condition.

There have been more than 400 kinds of ingredients in our data set, and the total number of images exceeds 60,000. Due to insufficient images for some categories, we currently do not include all of them in the data set for training. We will continue to collect images of ingredients to enlarge the data set further. In future work, more kinds of attention mechanisms will be tested to improve the feature extraction ability of the network, so that the generalization capability and performance in different data sets can also be improved. In addition, we will continue the study on food quality inspection.

## Figures and Tables

**Table 1:** Statistics of the food data set.

Data Source	Shooting Equipment	Resolution	Classes	Number of Pictures
Enterprise	Smart Catering System	800 × 600	320	8325
School	Smart Catering System	800 × 600	175	37,051
Market	Phones	1280 × 720	70	14,408
Internet	/	Uncertain	63	8853

**Table 2:** Accuracy at different resolutions.

**Resolution**	300 × 300	350 × 350	400 × 400	480 × 480	540 × 540	600 × 600	650 × 650	720 × 720
**Accuracy (%)**	72.36	76.89	77.46	80.10	80.45	81.73	79.30	76.12

**Table 3:** Open set performance.

Class	Images	Openness	Our Model Accuracy (%)	F1 (%)	Threshold Accuracy (%)
15	785	0.25	72.4	70.2	65.2
15	785	0.40	70.2	69.4	64.0
47	2100	0.21	71.8	70.5	66.3
60	2600	0.25	74.7	72.9	69.3

**Table 4:** Comparison of different models.

Model	Parameters (M)	Accuracy (%)	Precision (%)	Recall (%)	F1 (%)
Resnet50 [28]	25.5	73.51	74.30	72.88	73.23
Resnet101 [28]	44.55	77.23	77.37	77.67	77.16
Efficientnet-B1 [29]	7.80	79.75	79.86	78.80	79.02
DenseNet121	6.98	81.73	81.57	81.46	81.44
DenseNet161	26.5	74.43	76.16	73.07	74.40

**Table 5:** Performance of DenseNet with different attention modules.

Model	Parameters (M)	Accuracy (%)	Precision (%)	Recall (%)	F1 (%)
DenseNet121	6.98	81.73	81.57	81.46	81.44
DenseNet121-1	6.99	93.55	91.20	94.41	92.75
DenseNet121-2	7.02	92.67	92.91	92.46	92.59
DenseNet121-3	7.12	93.19	93.50	93.22	93.31
DenseNet121-12	7.03	76.44	77.41	76.72	76.97
DenseNet121-23	7.15	82.72	82.76	82.32	82.40
DenseNet121-34	7.25	86.39	86.30	85.83	85.99
DenseNet121-1234	7.29	85.86	86.26	86.06	86.15
DenseNet121-1-d	6.99	89.01	88.85	88.99	88.91
DenseNet121-1-lse	6.99	95.02	94.42	94.78	94.51
DenseNet121-12-lse-d	7.03	87.43	88.13	88.33	87.94
**Ours**	**6.99**	**95.90**	**96.33**	**96.35**	**96.33**

**Table 6:** Comparison between our method and related work in ingredients classification task.

Author	Method	Classes Number	Database Size	Kind	Normal Accuracy (%)
Rocha et al. [32]	Feature fusion	15	2633	Vegetables, fruits	95.00
Hou et al. [30]	Bilinear pooling	292	160,000	Vegetables, fruits	83.51
Zeng et al. [33]	Image saliency	26	/	Vegetables, fruits	95.60
Wu et al. [12]	Transfer learning	60	/	Vegetables	72.45
**Ours**	**Attention based** **DenseNet**	**170**	**31,200**	**Vegetables, meat,** **sea food, fruits**	**95.90**

## Data Availability

Restrictions apply to the availability of these data. Data was owned by Zhejiang Guotong Electric Engineering Co., Ltd. and are available from the authors with the permission of Zhejiang Guotong Electric Engineering Co., Ltd.
